# Efficacy of Delafloxacin against the Biothreat Pathogen Burkholderia pseudomallei

**DOI:** 10.1128/AAC.00736-21

**Published:** 2021-09-17

**Authors:** Sandra McCurdy, Erin Duffy, Mark Hickman, Stephanie Halasohoris, Steven D. Zumbrun

**Affiliations:** a Melinta Therapeutics, Morristown, New Jersey, USA; b Joint Program Executive Office for Chemical, Biological, Radiological and Nuclear Defense (JPEO-CBRND), Joint Product Manager CBRN Medical, Frederick, Maryland, USA; c U.S. Army Medical Research Institute of Infectious Diseases (USAMRIID), Frederick, Maryland, USA

**Keywords:** *Burkholderia*, biothreat, delafloxacin, fluoroquinolone

## Abstract

The *in vitro* activity and *in vivo* efficacy of delafloxacin were evaluated against the causative pathogen of melioidosis, Burkholderia pseudomallei. Delafloxacin MICs were determined by broth microdilution according to CLSI guidelines for 30 isolates of B. pseudomallei. The *in vivo* efficacy of delafloxacin was studied at a range of doses in a postexposure prophylaxis (PEP) murine model of melioidosis. Delafloxacin was active *in vitro* against B. pseudomallei (MIC_90_, 1 μg/ml). When the mice were dosed with 50 mg/kg body weight and 80 mg/kg body weight delafloxacin at both 16 and 24 h, greater survival was observed (90% to 100% survival) than with the 30-mg/kg-dosed mice (70% survival). All delafloxacin-treated cohorts contained no detectable B. pseudomallei in the spleens at the end of the study. This contrasts with ceftazidime 16- and 24-h administration, which had 40% and 20% survival, respectively. Complete clearance of infection was observed for most but not all surviving cohorts administered ceftazidime. In the mouse model of infection, survival curves for delafloxacin- and ceftazidime-treated animals at treatment start times of 16 and 24 h were statistically significant (*P* values of <0.0001). Estimated daily delafloxacin exposures in the B. pseudomallei murine aerosol study were similar to daily human exposures with the approved twice a day (BID) intravenous (i.v.) (300 mg) or oral (450 mg) dosing regimens. Based on its *in vitro* and *in vivo* activity, its safety, and its tolerability profile, delafloxacin may offer an attractive treatment option as PEP or eradication therapy for B. pseudomallei. Evaluation in other *in vivo* infection models for B. pseudomallei should be considered.

## INTRODUCTION

Burkholderia pseudomallei is an aerobic, non-spore-forming, nonfermenting Gram-negative intracellular pathogen that resides in soil, water, and plants in regions of endemicity (1). It is the causative agent of melioidosis, a serious disease that affects humans and other animal species, including sheep, goats, swine, horses, cats, dogs, and cattle (2). Most cases of melioidosis are found in southeast Asia and northern Australia; however, it has been found in all parts of the world. In the United States, it has been identified in travelers, immigrants, and military personnel returning from countries of endemicity (2).

The pathogenesis of melioidosis involves invasion by B. pseudomallei into host cells such as the epithelial cells in the respiratory mucosa or skin, depending upon the route of entry. The organism is then able to invade and propagate in multiple cell types, including phagocytic (monocytes, neutrophils, and macrophages) and nonphagocytic cells. The organism can survive within phagosomes and phagolysosomes within cells and enter the cytoplasm, where it is able to replicate (3).

Humans are believed to acquire the infection by inhalation of contaminated dust or water droplets, ingestion of contaminated water, and through skin contact with contaminated soil. Because inhalation of B. pseudomallei can lead to severe disease with high mortality, the U.S. Centers for Disease Control and Prevention (CDC) classifies B. pseudomallei as a category B biological threat agent. Clinical manifestations of melioidosis are diverse. Approximately 85% of cases are acute and include cutaneous infections such as abscesses, pulmonary and bloodstream infections, and disseminated infection (2). Approximately 11% of infections become chronic, while a small number of patients (4%) develop reactivation of pulmonary disease more than 10 years later (4). Mortality rates for patients with melioidosis vary from 9% to 70% depending upon the country (1).

Antibiotic treatment of B. pseudomallei infections depends upon patient presentation. Treatment usually includes an intensive phase for acute infection (parenteral ceftazidime, amoxicillin-clavulanic acid, or meropenem for 10 to 14 days or for as long as 8 weeks), followed by an eradication phase (oral trimethoprim-sulfamethoxazole for 3 to 6 months) (5). Postexposure prophylaxis (PEP) (oral trimethoprim-sulfamethoxazole or amoxicillin-clavulanate for 21 days) is recommended occasionally, for example, after accidental laboratory exposures (5).

Because of the intracellular penetration properties of the fluoroquinolone class of antibiotics, ciprofloxacin was used in two clinical trials for eradication treatment of melioidosis (6). In both trials, an unacceptably high failure rate was observed for ciprofloxacin compared to that of the comparator agent treatment, and as a result, regimens containing fluoroquinolones such as ciprofloxacin are not currently recommended for treating melioidosis (5, 7). Ciprofloxacin’s lack of efficacy was also observed in a murine inhalational murine model of melioidosis where antibiotic treatment was initiated at 6 or 24 h postchallenge and continued for 14 days. The survival of infected mice following 14 days of ciprofloxacin treatment was 0% for treatment initiated at 6 h and 0% for treatment initiated at 24 h in a 63-day observational study (8).

Delafloxacin differs from the other fluoroquinolones, such as ciprofloxacin, in the absence of a protonatable substituent, which confers to the molecule a weak acid character. This property further increases delafloxacin’s potency in acidic environments as found in abscesses or within the lysosomes or phagolysosomes of a phagocytic or nonphagocytic cell. Early studies with delafloxacin demonstrated intracellular accumulation and intracellular activity against Staphylococcus aureus in macrophages. Furthermore, this activity was increased at acidic pH relative to that at neutral pH (9). In addition, delafloxacin showed enhanced activity against Escherichia coli and Klebsiella pneumoniae in the acidic urinary environment (pH 5.0 to 6.0), whereas no enhanced activity was observed for ciprofloxacin (10). Therefore, these molecular properties may result in improved efficacy against B. pseudomallei, which can survive within host cell organelles, such as lysosomes, where the pH is acidic (11).

The spectrum of activity for delafloxacin includes Gram-positive, Gram-negative, and atypical pathogens, including activity against fluoroquinolone-nonsusceptible methicillin-resistant Staphylococcus aureus (MRSA) isolates (12). Delafloxacin offers the flexibility of intravenous (i.v.) and oral treatment, with no demonstrable QT-interval prolongation or phototoxicity (13, 14). It is U.S. Food and Drug Administration (FDA) and European Medicines Agency (EMA) approved for the treatment of patients with acute bacterial skin and soft tissue infections (ABSSSI) and for community-acquired bacterial pneumonia (CABP) (14).

Thus, the approved clinical indications, the lack of QT prolongation and phototoxicity, and the convenience of i.v. or oral formulation are attributes that may make delafloxacin a good option for PEP in a biothreat situation and/or as eradication treatment for B. pseudomallei infection. Therefore, both *in vitro* and *in vivo* efficacy using a whole-body aerosol challenge against B. pseudomallei data are presented here.

## RESULTS

### *In vitro* activity.

Using standard MIC testing conditions, delafloxacin was 4-fold more potent by MIC_90_ than the comparator agent ceftazidime against B. pseudomallei strains (Table 1) The delafloxacin and ceftazidime MIC values for the aerosol challenge strain. B. pseudomallei 1026b administered in the *in vivo* study were 0.25 μg/ml and 0.5 μg/ml, respectively. Since ciprofloxacin is not considered a standard treatment for melioidosis, it was not included as a comparator in the *in vitro* study.

**TABLE 1:** Delafloxacin and ceftazidime activity against B. pseudomallei^*a*^

Drug	MIC (μg/ml)		
	Range	50%	90%
Delafloxacin	0.12 to 2	0.5	1
Ceftazidime	0.5 to >64	4	4

a *N* = 30.

### *In vivo* activity.

**FIG 1:**
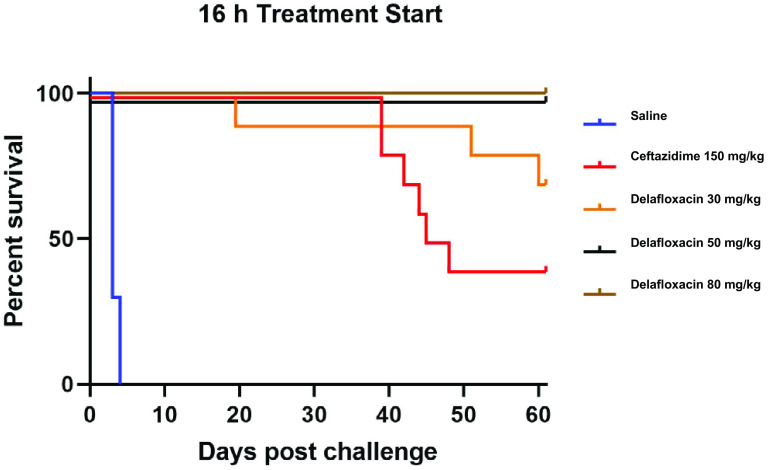
*In vivo* efficacy of ceftazidime and delafloxacin administered beginning 16 h after aerosol challenge with B. pseudomallei strain 1026b (135 ± 19 LD_50_) in BALB/c mice (*n* = 10) treated for 21 days.

**FIG 2:**
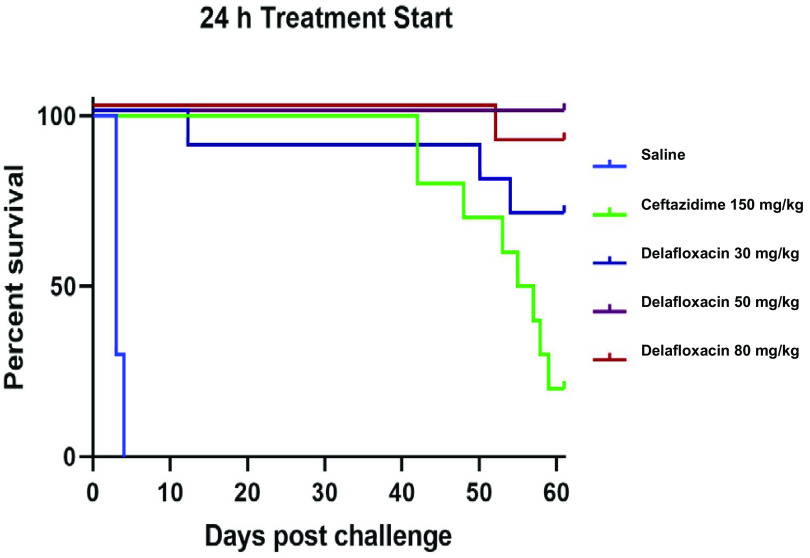
*In vivo* efficacy of ceftazidime and delafloxacin administered beginning 24 h after aerosol challenge with B. pseudomallei 1026b (135 ± 19 LD_50_) in BALB/c mice (*n* = 10) treated for 21 days.

The primary endpoint for this study was survival at day 62, which was also the time point when the study was terminated. The overall survival for all cohorts administered delafloxacin was 88%, while the ceftazidime-treated cohorts had an average survival of 30%, with an average mean time to death of 51 days. When the mice were dosed with 50 mg/kg body weight and 80 mg/kg body weight delafloxacin at both 16 and 24 h, greater survival was observed (90% to 100% survival) than for the 30-mg/kg dosed mice (70% survival). All delafloxacin-treated cohorts contained no detectable B. pseudomallei in the spleens at the end of the study. The lower limit of detection for this assessment was 1 × 10^2^ CFU/g spleen (Table 2). This contrasts with ceftazidime 16- and 24-h administrations, which had 40% and 20% survival, respectively. Complete clearance of infection was observed for most but not all cohorts administered ceftazidime. One mouse in the ceftazidime 16-h PEP treatment group had 1.17 × 10^7^ CFU/g of spleen (Fig. 1 and 2).

**TABLE 2:** Spleen counts for B. pseudomallei murine aerosol efficacy study (day 62)

Treatment	Mouse no.	Spleen wt (g)	B. pseudomallei CFU/spleen
Ceftazidime			
150 mg/kg, 24 h, q6h, 21 days	1	0.079	0
	2	0.102	0
150 mg/kg, 16 h, q6h, 21 days	1	0.099	0
	2	0.343	1.17 × 10^7^
	3	0.147	0
Delafloxacin			
30 mg/kg, 16 h, q6h, 21 days	1	0.094	0
	2	0.086	0
	3	0.093	0
50 mg/kg, 16 h, q6h, 21 days	1	0.086	0
	2	0.084	0
	3	0.093	0
80 mg/kg, 16 h, q6h, 21 days	1	0.084	0
	2	0.070	0
	3	0.108	0
30 mg/kg, 24 h, q6h, 21 days	1	0.099	0
	2	0.089	0
	3	0.102	0
50 mg/kg, 24 h, q6h, 21 days	1	0.090	0
	2	0.119	0
	3	0.115	0
80 mg/kg, 24 h, q6h, 21 days	1	0.091	0
	2	0.093	0
	3	0.088	0

To determine whether survival times between the delafloxacin- and ceftazidime-treated groups were statistically significant, the log rank test was assessed by GraphPad Prism, version 5.04. The test examined the linear trend between group and survival. Comparison of survival curves for treatment start times of 16 and 24 h were statistically significant, with *P* values of <0.0001.

## DISCUSSION

Delafloxacin demonstrated potent *in vitro* and *in vivo* activity against the category B biothreat pathogen, B. pseudomallei. Compared to ceftazidime, delafloxacin demonstrated 4-fold more potent *in vitro* activity against a geographically diverse set of B. pseudomallei isolates and 2-fold more potent activity against the aerosol challenge strain used in this study under normal testing conditions of pH 7.2. In addition, there was a statistically significant improvement in survival times compared to that with ceftazidime, suggesting that delafloxacin could be an effective PEP option. Notably, all cohorts in the delafloxacin group had complete splenic clearance of B. pseudomallei, while one mouse in the ceftazidime-treated cohort demonstrated a high bacterial load.

Several comparators could have been chosen for this study; however, ceftazidime is typically used to treat the acute phase of B. pseudomallei infection and is therefore an appropriate comparator for PEP due to its known potency and historical efficacy (15–17). Furthermore, treatment with 150 mg/kg ceftazidime every 6 h (q6h) is approximately 4 times the high-dose equivalent for treatment of the acute phase of melioidosis. This dosing has been used as a control for previous studies and was determined empirically to achieve ∼50% survival with treatment starting at 8 to 16 h postinfection following aerosol challenge at ∼100 times the 50% lethal dose (LD_50_).

In a separate study of an inhalational murine model of melioidosis, another fluoroquinolone antibiotic, finafloxacin, which, like delafloxacin, has increased potency at acid pH, was evaluated. In this study, antibiotic treatment was initiated at 6 or 24 h postchallenge and continued for 14 days, and the mice were observed for 63 days. The survival of infected mice following 14 days of finafloxacin treatment was 80% when treatments were initiated at 6 h and 60% when treatment was initiated at 24 h. Unlike delafloxacin, where overall survival was 88% and complete splenic clearance was observed, B. pseudomallei was not completely cleared from the spleens in both finafloxacin treatment arms (8).

Estimated daily delafloxacin exposures in the B. pseudomallei murine aerosol study were similar to daily exposures in humans with the approved twice a day (BID) i.v. (300 mg) or oral (450 mg) dosing. A pharmacokinetic assessment was not performed during the current study, and so estimated exposures were generated by performing simulations using a previously developed compartmental pharmacokinetic (PK) model based on mouse plasma PK data from several studies (data on file at Melinta Therapeutics, Morristown, NJ). At the dosages given in this study, the estimated steady-state areas under the concentration-time curves for the free drug from 0 to 24 h (AUC_0–24_) were 6.28, 10.4, and 16.5 h · μg/ml for the 30-, 50-, and 80-mg/kg subcutaneous doses given four times a day (QID), respectively. In humans, plasma steady-state free AUC_0–24_ values for i.v. 300 mg and oral (p.o.) 450 mg, each given BID, were 7.49 and 9.86 h · μg/ml, respectively (based upon plasma protein binding of 84%) (14). Therefore, human exposures at the approved i.v. and oral dosages are similar to exposures that were highly efficacious in mice.

Based on its *in vitro* and *in vivo* activity, its safety, and its tolerability profile, delafloxacin may offer an attractive treatment option as PEP or eradication therapy for B. pseudomallei. Since the primary site of infection for B. pseudomallei is the lungs, one limitation of this study was that the lungs were not collected for determination of colony counts on day 62. Evaluation in other *in vivo* infection models for B. pseudomallei should be considered.

## MATERIALS AND METHODS

### *In vitro* study.

The organisms used for susceptibility testing were obtained from the USAMRIID. This was a geographically biodiverse set of 30 strains of B. pseudomallei and included the aerosol challenge strain 1026b. All experiments presented in this study were carried out according to standard operating procedures that were assessed and approved by USAMRIID’s biosafety committee.

### Antimicrobial susceptibility testing.

MICs were determined by the microdilution method in 96-well plates, according to the Clinical and Laboratory Standards Institute (18). Antibiotics were serially diluted 2-fold in 50 μl of cation-adjusted Mueller-Hinton broth (CAMHB). The antibiotic ranges were 8 to 0.004 μg/ml or 64 to 0.03 μg/ml based on a final well volume of 100 μl after inoculation. Plates were incubated at 35°C, and MIC values were determined visually at 18 to 24 h. Quality control (QC) strains were tested concurrently and included E. coli ATCC 25922, S. aureus ATCC 29213, and Pseudomonas aeruginosa ATCC 27853. All QC results were within published ranges (19).

### Drug preparation.

The intravenous formulation of delafloxacin (Melinta Therapeutics, Morristown, NJ, USA) was used in the study. Reconstituted solutions were stable for up to 24 h. Liquid suspensions were prepared fresh in 0.9% saline irrigation (SI) daily and stored at 2 to 8°C. The volume of subcutaneous (SC) administrations was 0.2 ml.

The parenteral formulation of ceftazidime (Hospira, Lake Forest, IL, USA) was used in the study and was stored under refrigeration at 2 to 8°C.

### Aerosol infection model.

The whole-body aerosol challenge utilized the B. pseudomallei 1026b strain, obtained from the CDC, which was originally isolated in 1993 from a human case of melioidosis in Thailand. Aerosol was generated using a three-jet collision nebulizer (20). All aerosol procedures were controlled and monitored using an automated bioaerosol exposure system, operating with a whole-body rodent exposure chamber (21). Integrated air samples were obtained from the chamber during each exposure using an all-glass impinger (AGI).

The target LD_50_ for the aerosol challenge model in BALB/c mice is ∼100 LD_50_. This typically results in a survival rate of ∼50% by the end of the study period with a treatment start of 8 to 16 h. The amount of challenge material is estimated by culture prior to challenge by serial dilution on chocolate agar plates. The inhaled dose, CFU/mouse, of B. pseudomallei was estimated using Guyton’s formula (22). The mean inhaled dose was 135 (± 19)× LD_50_.

Specific-pathogen-free, female 6- to 8-week-old BALB/c mice (Charles River Laboratories, Frederick, MD, USA), weighing approximately 20 g, were infected in groups of 40. Mice were randomly placed into separate cages upon the conclusion of each aerosol administration to ensure each treatment group had equal numbers of mice for each aerosol exposure, since ≤50 mice can be exposed in a single aerosol run.

### Efficacy assessment.

To determine the efficacy of delafloxacin, a range of doses (30 to 80 mg/kg) was investigated as outlined. Delafloxacin was administered SC, beginning 16 and 24 ± 1 h postchallenge (PEP) and continued for a total of 21 days. The comparator for the study was ceftazidime (150 mg/kg), administered intraperitoneally (i.p.) (q6h) for 21 days, starting 16 and 24 ± 1 h after challenge. An untreated control group received 0.2 ml saline (0.9% SI) i.p. (q6h) for 21 days, starting 24 ± 1 h after challenge. Survival was assessed four times daily during treatment, and at least twice daily thereafter. Moribund animals were euthanized as necessary and counted as dead. In accordance with the accepted timeline for this animal model for melioidosis infection, the study was terminated at day 62. The longer observation period allowed for differentiation between “real” and “apparent” cure, as some bacterial diseases can result in a chronically infected state. At the conclusion of the observation period, all animals were humanely euthanized. In addition, upon study termination, spleens from three surviving mice from each cohort were excised, and the homogenates were plated (10^2^ to 10^4^) on chocolate agar plates incubated at 35°C for 24 h to determine the degree of B. pseudomallei infection.

### Study analysis.

All analyses were performed by employing a stratified Kaplan-Meier analysis with a log rank test as implemented in Prism, version 5.04 (GraphPad Software). Comparisons of survival curves for treatment start times of 16 and 24 h were statistically significant with *P* values of <0.0001.
